# Identification of pancreatic cancer invasion-related proteins by proteomic analysis

**DOI:** 10.1186/1477-5956-7-3

**Published:** 2009-02-14

**Authors:** Naomi Walsh, Norma O'Donovan, Susan Kennedy, Michael Henry, Paula Meleady, Martin Clynes, Paul Dowling

**Affiliations:** 1 National Institute for Cellular Biotechnology, Dublin City University, Glasnevin, Dublin 9, Ireland, UK; 2 St Vincent's University Hospital, Dublin 4, Ireland, UK

## Abstract

**Background:**

Markers of pancreatic cancer invasion were investigated in two clonal populations of the cell line, MiaPaCa-2, Clone #3 (high invasion) and Clone #8 (low invasion) using proteomic profiling of an *in vitro *model of pancreatic cancer.

**Materials and methods:**

Using 2D-DIGE followed by MALDI-TOF MS, two clonal sub-populations of the pancreatic cancer cell line, MiaPaCa-2 with high and low invasive capacities were incubated on matrigel 24 hours prior to analysis to stimulate cell-ECM contact and mimic *in vivo *interaction with the basement membrane.

**Results:**

Sixty proteins were identified as being differentially expressed (> 1.2 fold change and *p *≤ 0.05) between Clone #3 and Clone #8. Proteins found to have higher abundance levels in the highly invasive Clone #3 compared to the low invasive Clone #8 include members of the chaperone activity proteins and cytoskeleton constituents whereas metabolism-associated and catalytic proteins had lower abundance levels. Differential protein expression levels of ALDH1A1, VIM, STIP1 and KRT18 and GAPDH were confirmed by immunoblot. Using RNAi technology, STIP1 knockdown significantly reduced invasion and proliferation of the highly invasive Clone #3. Knockdown of another target, VIM by siRNA in Clone #3 cells also resulted in decreased invasion abilities of Clone #3. Elevated expression of STIP1 was observed in pancreatic tumour tissue compared to normal pancreas, whereas ALDH1A1 stained at lower levels in pancreatic tumours, as detected by immunohistochemistry.

**Conclusion:**

Identification of targets which play a role in the highly invasive phenotype of pancreatic cancer may help to understand the biological behaviour, the rapid progression of this cancer and may be of importance in the development of new therapeutic strategies for pancreatic cancer.

## Background

Pancreatic cancer is the tenth most common cancer in Europe, and accounts for approximately 2.5% of cancer in males and females [1]. The median survival is 8–12 months for patients presenting with locally advanced and unresectable disease, and only 3–6 months for those with metastatic disease at presentation [2]. Surgery offers the best curative treatment, but < 15% of patients present with tumours eligible for resection at initial diagnosis due to aggressive local and perineural invasion, early metastasis to liver and lymph nodes, formation of desmoplastic stromal reaction within the tumour and resistance to chemotherapy and radiation [3]. Therefore, there is an urgent need to develop molecular diagnostic biomarkers and targets to detect pancreatic cancer at an earlier stage, which may help to improve treatment and survival of pancreatic cancer patients. The development of invasive and metastatic pancreatic cancer is complex and poorly understood. Metastasis is defined as the ability of tumour cells at the primary site to invade local tissue, cross the basement membrane and tissue barriers and re-establish at distant secondary locations. This process of metastasis is not random. A cascade of complex interactions between the cancer cell and its surroundings results in the metastatic cascade; Tumour cells must first break signalling contact with neighbouring cells, degrade and penetrate the basement membrane and then invade the interstitial stroma in order to reach blood/lymph vessels [4]. Intravasation requires penetration of the blood/lymph systems. The tumour cells must then exit the lymph system or blood stream at a new site (extravasation) and proliferate in the secondary organ [5]. The heterogeneous nature of some tumours is associated with sub-populations of highly metastatic tumour cells existing at very early stages of primary tumour development [6]. We previously isolated clonal sub-populations of the human pancreatic cancer cell line, MiaPaCa-2 through serial dilution. Two of these sub-clones, Clone #3 and Clone #8, displayed altered malignant properties. Clone #3 showed higher invasion with low levels of adhesion, while Clone #8 displayed decreased invasion with increased adhesion to ECM proteins. Clone #8 was sensitive to anoikis, and displayed low colony-forming efficiency in an anchorage-independent growth assay compared to Clone #3. This model provides a unique *in vitro *representation of an invasive pancreatic carcinoma. The aim of this study was to identify novel proteins involved in pancreatic cancer invasion using an *in vitro *model of pancreatic cancer invasion. Protein expression of Clone #3 (highly invasive) and Clone #8 (low level of invasion) were compared using 2D-DIGE followed by MALDI-TOF-MS for protein profiling.

## Results

### Invasion assays

**Figure 1:**
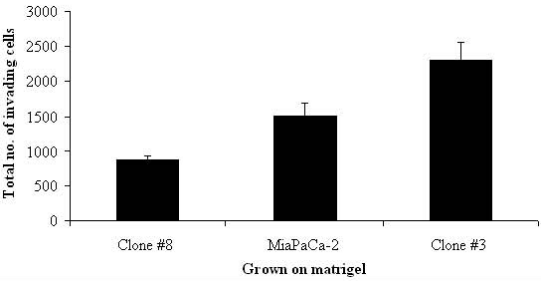
**Graph displays the total number of cells invading after 24 hr incubation on matrigel of Clone #8, MiaPaCa-2 and Clone #3**. Experiments performed in triplicate.

Two clonal populations, Clone #3 and Clone #8 were isolated from the human pancreatic cancer cell line, MiaPaCa-2. Clone #3 is highly invasive compared to the parent MiaPaCa-2, whilst Clone #8 displays distinctively less invasion than the parent as shown by the total number of cells invading after preincubation on matrigel for 24 hrs (Fig 1).

### Identification of proteins by 2D-DIGE analysis

**Figure 2:**
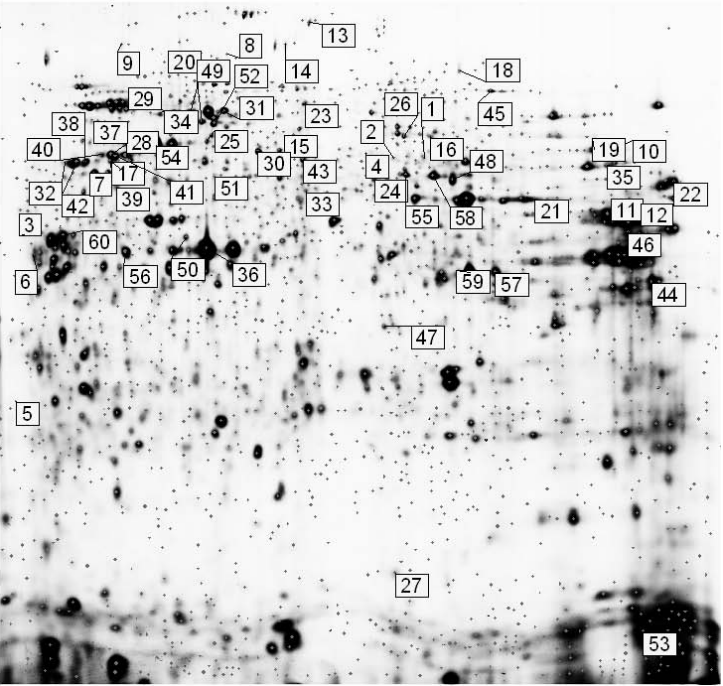
**Representative 2D DIGE gel image of Cy2-labelled pool of Clone #3 and Clone #8 cell lysate samples**. Differentially expressed proteins that have been successfully identified by MALDI-TOF MS (*p *≤ 0.05, protein fold ≥ 1.2) are represented on the gel using DeCyder software. Proteins are labelled numerically for visual clarity and are outlined in Table S1.

To investigate proteins potentially involved in invasion in this model for human pancreatic cancer, we systematically analysed protein expression from Clone #3 and Clone #8 grown on matrigel 24 hours prior to protein extraction using 2D-DIGE. Biological variation analysis of spots showing greater than 1.2-fold change in expression with a *t*-test score of less than 0.05, revealed 60 differentially expressed proteins between Clone #3 and Clone #8 (Fig 2 and Table S1 Additional file 1). Table S1 is a list of the differentially expressed proteins with their protein accession numbers, % coverage, theoretical pI, and molecular weight, calculated fold change, *P *values and function-(Human Protein Reference Database (HPRD)) . Theoretical pI and MW provides information not only on full-length protein expression, but expression of modified, splice variant, cleavage product, and processed proteins. Any protein modification that leads to a change in overall protein charge and/or molecular weight (MW) will generate a different spot on the 2D gel. Modification specific staining can identify whether a specific post-translational modification is responsible for the shift, and mass spectrometry can potentially identify the source of isoelectric point (pI) and/or MW differences.

Of the 60 proteins identified, 49 of these proteins were found in higher abundance and 11 were expressed at lower levels in the highly invasive Clone #3. Many highly expressed proteins in Clone #3 correspond to the cytoskeleton (vimentin, vinculin, tubulin alpha-6, beta-tubulin, alpha-tubulin and gamma-actin), the chaperone family of proteins (heat shock proteins, KIAA0098, stress-induced phosphoprotein 1 and MTHSP75). Other highly abundant proteins included some associated with translation and transport, receptor signalling and ligase activity. Among the 11 low abundant proteins, most are involved in the catalytic/glycolysis activity. Keratin 18 (KRT18), a cytoskeletal protein is 2.9-fold less abundant in Clone #3 cells, and low expression of KRT18 has been previously implicated in a more aggressive phenotype [7].

### Gene ontology enrichment analysis

**Figure 3:**
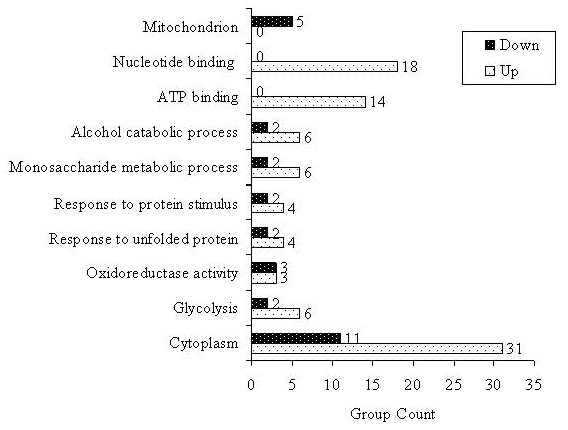
**Term-ranking Gene Ontology categories**. Representation of the 10 top-ranked functional categories, using GO terms that are enriched in all significantly differentially expressed proteins between Clone #3 versus Clone #8.

Using DAVID gene ID tool software , all the proteins differentially expressed in our model were converted to their gene IDs. Gene ontology (GO STAT)  was then used to classify the proteins and their corresponding genes into gene categories and assign functional categories. Enrichment of a particular ontology term, for significantly expressed genes in response to the process under study, means that the ontology term is likely to be involved in the process. In our study, the process refers to invasion in pancreatic cancer. Using the over-expression function of the software and false discovery rate (Benjamini) stats, 52 GO terms were found significantly enriched between Clone #3 versus Clone #8. The molecular functions of the proteins identified in this study were classified according to GO database. Figure 3 displays the top 10 GO biological process categories of invasion-related differentially expressed proteins in our pancreatic cancer model. Profiles based on differentially expressed proteins showed clear differences between the two cell lines. For example, the "cytoplasm" term achieved the highest degree of significance in the up-regulated gene class (*p *= 1.40E-09), with "glycolysis" (*p *= 4.05E-07) and "nucleotide binding" (*p *= 1.04E-06) also highly significant terms. In the down-regulated class, "oxidoreductase activity" (*p *= 0.0006), "aldehyde dehydrogenase activity" (*p *= 0.003) and "mitochondrion" (*p *= 0.03) were also significantly enhanced.

### Confirmation of identified proteins by immunoblot analysis

**Figure 4:**
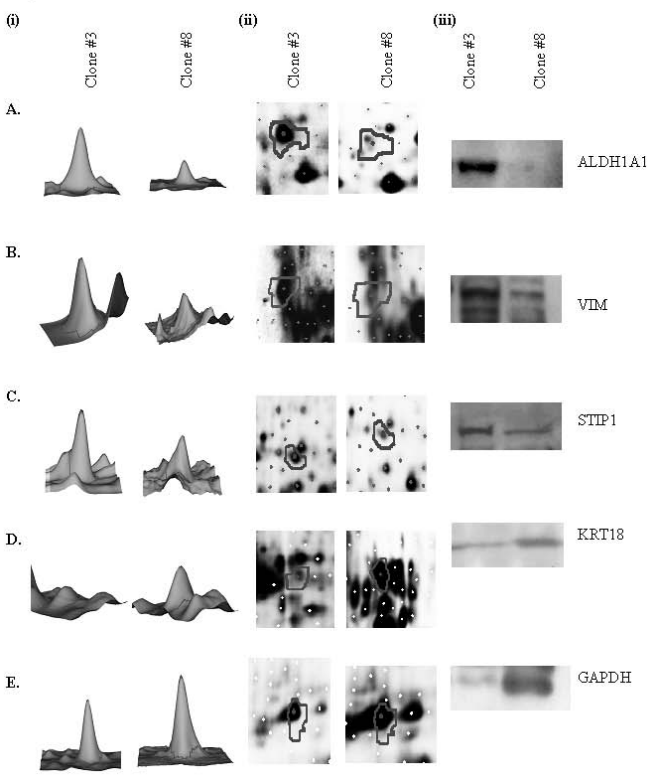
**Differentially expressed proteins, A. ALDH1A1, B. VIM, C. STIP1, D. KRT18 and E. GAPDH in Clone #3 versus Clone #8 confirmed by (i) 3D spot image, (ii) PEM spot and (iii) immunoblot**.

Immunoblot analysis was carried out to confirm the differential expression observed for A. ALDH1A1, B. VIM, C. STIP1, D. KRT18 and E. GAPDH in Clone #3 and Clone #8. In all cases, the results were consistent with proteomic analysis. Figure 4 (A-E) details the high and low abundance of proteins by (i) 3D spot image, (ii) protein expression map and (iii) immunoblot in the comparison of Clone #3 versus Clone #8.

### A novel role for stress-induced phosphoprotein 1 (STIP1) in invasion

**Figure 5:**
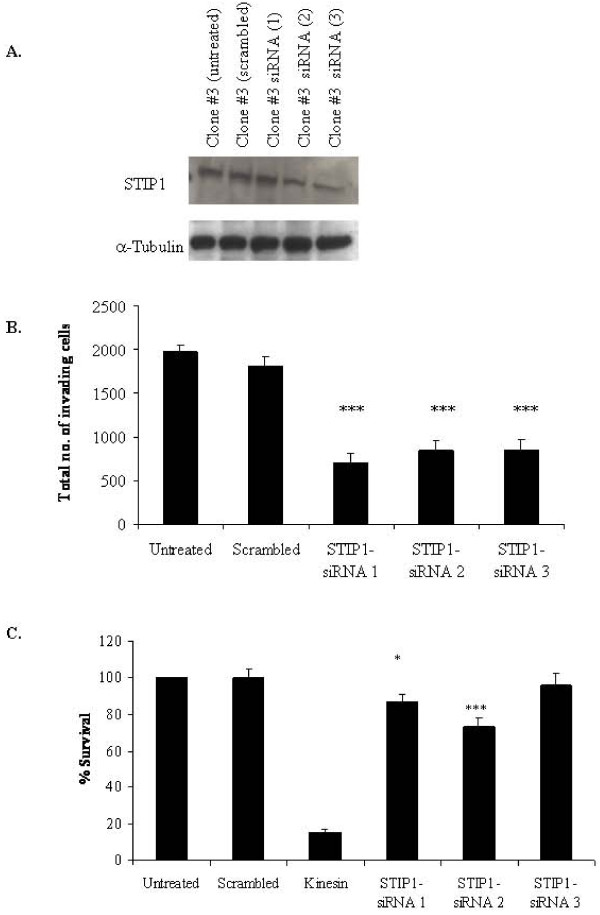
**A. Immunoblot of STIP1 silencing in Clone #3 cells untreated control, scrambled control, STIP1-siRNA (1), STIP1-siRNA (2) and STIP1-siRNA (3) (*upper*) and α-tubulin as loading control (*lower*)**. **B**. Total number of invading cells after siRNA transfection in Clone #3 untreated control, scrambled control, STIP1-siRNA (1), STIP1-siRNA (2) and STIP1-siRNA (3). Results are displayed as the total number of invading cells, determined by counting the number of cells per field in 10 random fields, at 200× magnification. The average number of cells per field was then multiplied by a factor of 140 (growth area of membrane/field area viewed at 200× magnification (calibrated using a microscope graticule)). Experiments performed in triplicate. Total mean number of cells invading at 200× magnification (*n *= 3). **C**. Proliferation assays of Clone #3 untreated control, scrambled control, STIP1-siRNA (1), STIP1-siRNA (2) and STIP1-siRNA (3). Results displayed as percentage survival relative to untreated control. Student's *t*-test; *p *= 0.05*, 0.01**, 0.005*** (*n *= 3).

STIP1 expression levels were 2-fold higher in Clone #3 compared to Clone #8. Invasion assays were carried out on untreated Clone #3 cells, cells treated with scrambled siRNA and three independent siRNAs for STIP1 (Fig 5A and 5B). STIP1 siRNA transfection significantly reduced the invasion of Clone #3 cells (3-fold (*p *= 0.0002) with STIP1-siRNA (1), 2-fold (*p *= 0.0002) with STIP1-siRNA (2) and 2-fold (*p *= 0.0003) with STIP1-siRNA (3). STIP1 had no effect on adhesion (data not shown), however, transfection of Clone #3 cells with STIP1-siRNAs decreased proliferation by 13% (*p *= 0.04) with STIP1-siRNA (1) and 27% (*p *= 0.003) with STIP1-siRNA (2). STIP1-siRNA (3) did not alter the proliferation of the cells (Fig 5C).

### Vimentin (VIM) involvement in the invasion of pancreatic cancer cells

**Figure 6:**
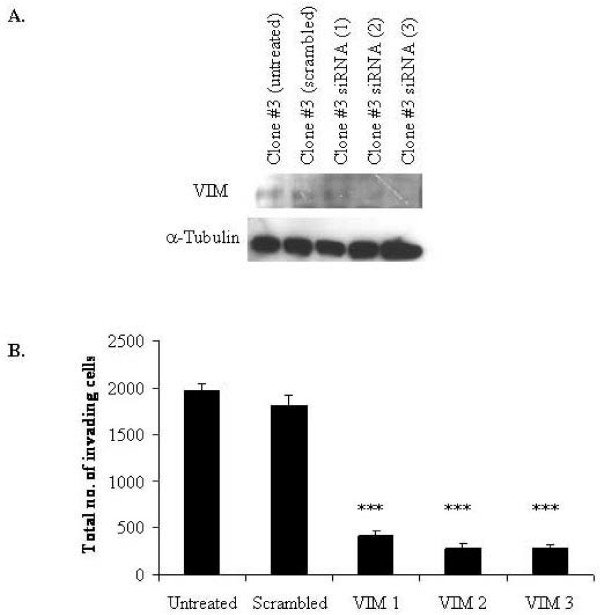
**A. Immunoblot of VIM knockdown in Clone #3 untreated control, scrambled control, VIM-siRNA (1), VIM-siRNA (2) and VIM-siRNA (3) (*upper*) and α-tubulin as loading control (*lower*)**. **B**. Invasion assay of Clone #3 cells after siRNA silencing of VIM. The total number of invading cells was determined by counting the number of cells per field in 10 random fields, at 200× magnification. The average number of cells per field was then multiplied by a factor of 140 (growth area of membrane/field area viewed at 200× magnification (calibrated using a microscope graticule)). Experiments performed in triplicate. Statistics; * ≤ 0.05, ** ≤ 0.01, *** ≤ 0.005.

VIM was detected at 5.5-fold higher levels in Clone #3 compared to Clone #8. Transfection of VIM-siRNAs reduced the expression of VIM in Clone #3 cells (Fig 6A). Invasion of VIM-siRNA transfected Clone #3 cells reduced invasion 4-fold (*p *= 0.00036) with VIM-siRNA (1), 6-fold (*p *= 0.00031) with VIM-siRNA (2) and 6-fold (*p *= 0.0004) with VIM-siRNA (3) (Fig 6B).

### Immunohistochemical (IHC) analysis of STIP1 and ALDH1A1 in pancreatic tissue

**Figure 7:**
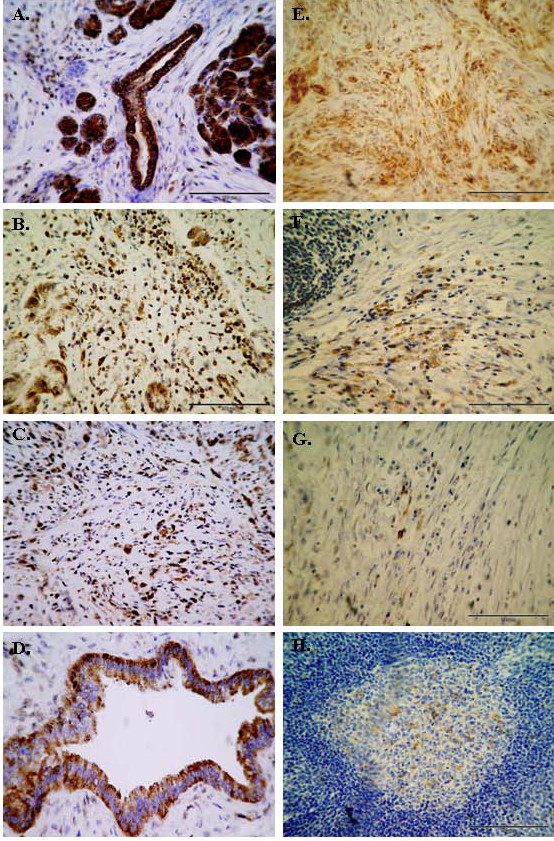
**IHC detection of STIP1 (A-D) and ALDH1A1 (E-H) in pancreatic cancer and normal pancreas tissues**. **(A) **Strong STIP1 cytoplasmic staining in PC tumour ducts. **(B-C) **Strong STIP1 expression in poorly differentiated PC tumours. **(D) **Moderate staining of normal pancreas ducts and acinar cells. **(E) **ALDH1A1 highly expressed in well differentiated PC tumour. **(F-G) **Weak ALDH1A1 staining observed in < 10% of poorly differentiated PC tumours. **(H) **Positive staining in epithelial cells of normal pancreas. Original magnification 200×.

IHC analysis was performed on pancreatic cancer (PC) tissue (n = 5) and corresponding normal pancreas (NP) tissue specimens (n = 5). IHC was used to validate the expression patterns of two proteins, STIP1 and ALDH1A1. In all cases STIP1 exhibited strong cytoplasmic staining in 5/5 PC specimens, concentrating strongest in areas of perineural invasion and necrotic cells in the epithelium (Fig 7A–C). However, STIP1 expression was also observed in the normal ductal and acinar cells of the exocrine pancreas (Fig 7D). STIP1 also stained surrounding duodenum, indicating that STIP1 may not be specific to the pancreas, but may have potential as a target for invasive cancer. Overall, increased STIP1 expression was observed in the tumour compared to normal tissues (Fig 7A–D). Strong ALDH1A1 expression was observed in 2/5 PC (Fig 7E). This may be associated with differentiation status, as strong ALDH1A1 expression was exclusive to well differentiated tumours (Table 1). Three moderate-poorly differentiated pancreatic cancer samples exhibited lower levels (< 10%) of ALDH1A1 (Fig 7F–G). ALDH1A1 staining was also observed in normal pancreas (Fig 7H), including islet cells.

**Table 1:** Type of pancreatic cancer included in the study

	Sex	Age	Tumour Type	Tumour Grade	Lymph node status
PC-01	M	48	Adenocarcinoma, ductal	Moderately differentiated	+ve
PC-02	F	43	Adenocarcinoma	Well differentiated	-ve
PC-03	F	67	Adenocarcinoma	Well differentiated	-ve
PC-04	M	36	Adenocarcinoma	Poorly differentiated	+ve
PC-05	M	67	Squamous cell	Poorly differentiated	No nodes

## Discussion

In this study, clonal sub-populations of the human pancreatic cancer cell line, MiaPaCa-2 were established and their invasion status assessed. Pancreatic cancer is characterised by early invasion and metastasis. As the invasive and metastatic cascade involves the interaction, attachment and degradation of the basement membrane *in vivo*, we allowed the cells grow on matrigel for 24 hours prior to proteomic analysis, to mimic the cells contact with the basement membrane. In this study, sixty proteins were identified as differentially expressed between Clone #3 and Clone #8 by 2D DIGE followed by MALDI-TOF MS. Bio-informatic profiling of the proteins identified was performed using GO (gene ontology). The GO STAT profiling of the differentially abundant proteins between Clone #3 and Clone #8 provided overall analysis of molecular functional changes in these cells. GO STAT analysis showed that proteins of higher abundance in this study are more related to the cytoplasm, ATP and nucleotide binding, while some proteins of lower abundance were exclusively classified as involved in the mitochondrion, suggesting loss of special organelle functions.

Three novel proteins, ALDH1A1, STIP1 and VIM were chosen to validate and further investigate their involvement in pancreatic cancer invasion.

ALDH1A1 was expressed at higher levels in the more invasive, Clone #3. ALDH1A1 is an enzyme involved in the conversion of aldehydes to their corresponding acids by NAD (P)^+ ^dependent reactions [8]. ALDH1A1 activity in cancer has been found to be responsible for resistance to oxazaphosphorines such as cyclophosphamide [9,10] and is involved in the irreversible oxidization of retinal to retinoic acid (RA) [11], which has been associated with invasion and adhesion in pancreatic cancer cell lines [12,13]. Expression of ALDH1A1 was shown to be up-regulated in the highly malignant ovarian cancer cell line, TOV-112D compared to the low malignant TOV-81D [14]. ALDH1A1 expression has been linked to more aggressive tumours suggesting a possible role in the invasive/drug resistance pathways.

Proteins involved in folding, stress response and degradation were also identified as important in our model. Molecular chaperones and folding enzymes are responsible for protein folding. Stress-induced phosphoprotein 1 (STIP1) was + 2.0-fold expressed in the highly invasive Clone #3 compared to Clone #8 (low invasion). STIP1 (Hsp70/Hsp90-organising protein (Hop)) mediates the association of the molecular chaperones Hsp70 and Hsp90 [15]. Heat shock proteins, Hsp90 and Hsp70 have previously been implicated in pancreatic cancer [16,17]. Eustace *et al*. [18] showed that Hsp90α chaperone-complex interactions are involved in MMP-2 activity and invasiveness in the fibrosarcoma cell line, HT-1080. STIP1 expression, through survival pathways and MMP activation, could contribute to invasion in the highly invasive Clone #3 pancreatic cancer cell line, therefore making it a potentially valuable target for pancreatic cancer therapy. Recently Sun *et al. *[19] confirmed the over expression of STIP1 (HOP) in hepatocellular carcinoma (HCC) by 2D fluorescence DIGE proteomic analysis. STIP1 is secreted by and shown to induce proliferation in glioma tumour cells through MAPK and P13 pathways [20].

VIM is a component of intermediate filaments (IF) of the cytoskeleton and is important in cell motility and movement, maintaining cell shape, integrity of the cytoplasm and stabilising cytoskeletal interactions. Proteomic analysis revealed an increased expression of VIM in the highly invasive Clone #3 compared to the low invasive Clone #8. Reduction of VIM expression by siRNA decreased the invasion of Clone #3 revealing a role for VIM in pancreatic cancer cell invasion. Many studies have shown VIM to be substantially expressed in liver metastases of pancreatic tumours [21], and expression is associated with increased invasiveness and metastasis potential for epithelial breast carcinoma [22], hepatocellular carcinoma [23] and cervical carcinoma [24]. Co-expression of VIM and cytokeratins (CKs) is associated with a more aggressive and metastatic phenotype in breast cancer [22,25]; however our data demonstrates increased expression of VIM with an associated decrease in cytokeratin 18 levels in Clone #3. Singh *et al*. [26] found that VIM expression contributed to the invasive phenotype of prostate cancer cell lines but could function at later stages of the invasive process. The high expression levels of VIM in our invasive cell line could represent a marker for the epithelial to mesenchymal transition (EMT), in agreement with previous pancreatic cancer studies [27,28]. Therefore, expression of VIM could be a confirmatory marker for EMT to a more aggressive phenotype. Javle *et al*. [29] found that EMT correlates with the activation of PI3 kinase and Ras/Erk pathways, which are known to be involved in invasion. Loss of VIM expression, up-regulation of adhesion proteins and reduced aggressiveness of *in vitro *invasion is associated with KRT18 expression [7]. However, conflicting reports have implicated KRT18 expression in carcinogenesis metastatic hepatocellular carcinoma (HCC) tissue [30] and as a predictive marker for lymph node metastasis in esophageal squamous cell cancer [31].

## Conclusion

In summary, knockdown of STIP1 and VIM by siRNA resulted in decreased invasion and proliferation (STIP1) in the highly invasive pancreatic cancer cell line, Clone #3. Expression of STIP1 and ALDH1A1 in pancreatic tissue was investigated using IHC analysis of pancreatic tumour and normal tissue. This is the first time that STIP1 and ALDH1A1 have been implicated in invasion and investigated in pancreatic tissue. Our results showed increased expression of STIP1 in ductal and highly invasive adenocarcinoma cells in the tumours with weaker positive staining in normal pancreas ducts and acinar cells. Staining of ALDH1A1 was mainly concentrated to < 10% of tumour cells in 3/5 poorly differentiated PC specimens. ALDH1A1 expression was also observed in the stromal and epithelial cells of the normal pancreas.

The proteomic profiling of pancreatic cancer cell lines with different invasion status from the same genetic background could help to elucidate the molecular mechanisms of pancreatic cancer invasion and may represent an *in vitro *model for pancreatic metastasis. The proteins identified in this study as involved in pancreatic cancer cell invasion may have potential as novel therapeutic targets and tumour markers of pancreatic cancer. However, the implication of these unique proteins identified as potential candidates associated with highly invasive pancreatic cancer needs to be fully evaluated. A large clinical study including serum samples, tissue and pancreatic juice, would be required in order to identify these proteins as potentially useful for diagnosis, staging, prognosis and response to therapy of pancreatic cancer.

## Methods

### Cell lines

The human pancreatic cell line MiaPaCa-2 was obtained from the European Collection of Cell Cultures (ECACC, UK). Clone #3 and Clone #8 were obtained by single cell dilution in this laboratory. Briefly the parental cell line was diluted to a concentration of 3 cells/ml and 100 μl plated onto each well of a 96-well plate. After 24 hours each well was studied for single cells, and allowed to grow into colonies. The colonies were then screened by invasion assay to assess their invasive abilities. Cells were maintained in a humidified atmosphere containing 5% CO_2 _at 37°C in DMEM supplemented with 5% FCS (Sigma-Aldrich). Antibiotics were not used in the growth media. All cell lines were free from Mycoplasma as tested with the indirect Hoechst staining method.

### Preincubation of cells with matrigel coated flasks

Matrigel (Sigma-Aldrich, UK) was coated onto flasks (1 ml/25 cm^2^) at a concentration of 1 mg/ml. The coated flasks were then placed at 4°C overnight. The flasks were placed into an incubator at 37°C for approximately 2 hrs to allow the matrigel polymerise. The excess media in the flasks was then removed and fresh complete media containing the cell suspension was added. Cells attached to the matrigel on the bottom of the flask and after 24 hrs were removed with 0.5 ml/T25 cm^2 ^dispase (BD Biosciences). Dispase is a bacillus derived neutral metaloprotease that recovers cells cultured on matrigel.

### Invasion assays

Invasion assays were performed as previously described [32]. 100 μl of matrigel (1 mg/ml) was placed into each invasion insert (Falcon) (8.0 μm pore size) in a 24 well plate (Costar). The coated inserts were incubated overnight at 4°C. Matrigel was allowed polymerize at 37°C for 1 hr, then washed with serum-free DMEM. 100 μl of fresh DMEM containing 5% serum was added to the wells and 1 × 10^5^/100 μl cells were seeded onto the insert. 500 μl of fresh DMEM with 5% serum was added to the well. After 24 hour incubation, the inside of the insert was wiped with a wet cotton swab. The under surface was gently rinsed with PBS and stained with 0.25% crystal violet for 10 minutes, rinsed again with sterile water and allowed to dry. To determine total number of invading cells, the inserts were then viewed under the microscope and the number of cells per field in 10 random fields were counted at 200× magnification. The average number of cells per field was then multiplied by a factor of 140 (growth area of membrane/field area viewed at 200× magnification (calibrated using a microscope graticule)). The mean values were obtained from a minimum of three individual experiments and were subjected to *t*-tests.

### Sample preparation and protein labelling

Cells at approximately 80% confluency were washed twice in PBS, twice in sucrose buffer before lysing in buffer containing 4% w/v CHAPS, 7 M urea, 2 M thiourea, 10 mM Tris-HCL, 5 mM magnesium acetate pH 8.5, and then homogenized by passing through a 25-gauge needle six times. Insoluble material was removed by centrifugation at 14000 rpm for 20 min at 10°C. Protein concentration was determined using the BCA protein assay kit (Bio-Rad). 50 μg of each biological repeat lysate was labelled with Cy3 and Cy5 (200 pmol) in the dark for 30 min and quenched with 50-fold molar excess of free lysine-to-dye. Samples were reverse-labelled in order to enable all comparisons and eliminate any dye-labelling bias. Reverse-labelling allows one to differentiate between sample-dependent differences and rare dye-dependent differences.

Samples were mixed and run on the same gels with an equal amount (50 μg) of Cy2-labeled standard. Cy2 was used as a standard on all gels to aid image matching and cross-gel statistical analysis [33].

### Protein separation by 2-DE and gel imaging

Immobilised 24 cm linear pH gradient (IPG) strips, pH 3–11, were rehydrated in rehydration buffer (7 M urea, 2 M thiourea, 4% CHAPS, 0.5% IPG buffer, 50 mM DTT) overnight, according to manufactures guidelines. IEF was performed using as IPGphor apparatus (GE Healthcare) for 40 kV/h at 20°C with resistance set at 50 mA. Strips were equilibrated for 20 min in 50 mM Tris-HCL, pH 8.8, 6 M urea, 30% v/v glycerol, 1% w/v SDS containing 65 mM DTT and then for 20 min in the same buffer containing 240 mM iodoacetamide. Equilibrated IPG strips were transferred onto 18 × 20 cm 12.5% uniform polyacrylamide gels poured between low fluorescence glass plates. Strips were overlaid with 0.5% w/v low melting point agarose in running buffer containing bromophenol blue. Gels were run at 2.5 W/gel for 30 min and then 100 W total at 10°C. All the images were collected on a Typhoon 9400 Variable Mode Imager (GE Healthcare). Statistics and quantification of protein expression were carried out in DeCyder software (GE Healthcare).

### Spot digestion and MALDI-TOF analysis

Excision of protein spots, trypsin digestion and protein identification by MS analysis using an Ettan MALDI-TOF Pro (GE Healthcare) was performed. Preparative gels containing 300 μg of protein were fixed in 30% v/v methanol, 7.5% v/v acetic acid overnight and washed in water, and total protein was detected by post-staining with CBB and Deep purple stain (Molecular Probes) for 3 hrs at room temperature. Excess dye was removed by washing twice in water, and gels were imaged using a Typhoon 9400 Variable Mode Imager (GE Healthcare) at the appropriate excitation and emission wavelengths for the stain. The subsequent gel image was imported into the BVA module of DeCyder software and was matched to images generated from DIGE analysis. Spots of interest were selected and confirmed using this software for subsequent picking using an Ettan Spot Picker. Gel plugs were placed into a presiliconised 1.5 ml plastic tube for destaining, desalting and washing steps. The remaining liquid above the gel plugs was removed and sufficient ACN was added in order to cover the gel plugs. Following shrinkage of the gel plugs, ACN was removed and the protein containing gel pieces were rehydrated for 5 min with a minimal volume of 100 mM ammonium bicarbonate. An equal volume of ACN was added, and after 15 min of incubation the solution was removed from the gel plugs and the samples were dried for 30 min using a vacuum centrifuge. Individual gel pieces were then rehydrated in digestion buffer (12.5 ng trypsin per μl of 10% ACN, 40 mM ammonium bicarbonate) to cover the gel pieces. Exhaustive digestion was carried out overnight at 37°C. After digestion, the samples were centrifuged at 12000 × g for 10 min using a bench top centrifuge. The supernatant was carefully removed from each sample and placed into clean plastic tubes. Samples were stored at -80°C until analysed by M.S. For spectrometric analysis, mixtures of tryptic peptides from individual samples were desalted using Millipore C-18 Zip-Tips (Millipore) and eluted onto the sample plate with the matrix solution (5 mg/ml CHCA in 50% ACN/0.1% TFA v/v). Mass spectra were recorded using the MALDI-TOF instrument operating in the positive reflectron mode at the following parameters: accelerating voltage 20 kV; and pulsed extraction; on (focus mass 2500). Internal calibration was performed using anti-analysis peaks at *m/z *842.50, *m/z *2211.104 and external calibration was performed using Pep4 mix. The mass spectra were analysed using MALDI evaluation software (GE Healthcare), and protein identification was achieved with the PMF Pro-Found search engine. An expectation value of < 0.002 was used for all reported identifications, which indicates a 0.2% chance the identification is random.

### Immunoblotting

Whole protein was extracted from cell lysates using 1× lysis buffer (50 mM Tris-Cl, 150 mM NaCl, and 0.5% NP-40). Lysates were centrifuged for 10 min at 14,000 rpm at 4°C. Protein concentrations were determined using the Bio-Rad protein assay (Bio-Rad). 35 μg of protein was separated by 7.5% and 15% SDS-PAGE under reducing conditions. Proteins were transferred to nitrocellulose membrane, efficiency and equal loading of protein was visualised by Ponceau S staining. Membranes were blocked at 4°C overnight in TBS (25 mM Tris-HCl, pH 7.4, 150 mM NaCl, 2.7 mM KCl) containing 5% (w/v) low fat milk powder. Membranes were probed with monoclonal antibodies, anti-aldehyde dehydrogenase (Abcam), anti-stress-induced phosphoprotein 1 (Santa Cruz) (Abcam), anti-cytokeratin 18 (Santa Cruz Biotechnology), anti-vimentin (Sigma), anti-GAPDH (Applied Biosystems). Secondary antibodies, anti-mouse, anti-rabbit and anti-goat were obtained from Sigma. Protein bands were detected with Luminol reagent (Santa Cruz Biotechnology).

### siRNA transient transfections

Three pre-designed STIP1 and VIM-siRNAs (Ambion) were chosen and transfected into cells. For each set of siRNA transfections carried out, a control (non-transfected) and a scrambled (SCR) siRNA transfected control were used. siRNA experiments were set up using 2 μl NeoFx to transfect 30 nM siRNA in a cell density of 3 × 10^5 ^per well of a 6-well plate. Transfection medium was removed after 24 hours and replaced with fresh growth medium. The transfected cells were collected for immunoblot and assayed for changes in invasion capacity at 48 hours using the *in vitro *invasion assay (as previously described).

### IHC Analysis

#### Patients

The patient group consisted of 5 consenting patients diagnosed with primary tumours of the pancreas. All patients were treated at St. Vincent's University Hospital (SVUH), Dublin in 2005. IHC studies on tumour-free pancreatic tissue were performed using corresponding non-cancerous tissue. Pathological material was examined on each case by SK. Formalin-fixed paraffin-embedded pancreatic tumour tissue and corresponding normal pancreas was available for all patients. Representative 4-μm sections of tissue block were cut using a microtome, mounted onto poly-l-lysine coated slides and dried overnight at 37°C. Slides were stored at room temperature until required.

#### Immunohistochemistry

Briefly the slides were immunohistochemically stained using primary antibodies specific for ALDH1A1 and STIP1 from Abcam. The staining procedure includes an antigen retrieval step consisting of 20-minute incubation in pH 9.0 buffer (TARGET Retrieval, Dako) in a 95°C water bath followed by cooling to room temperature. Staining was performed using an automated staining apparatus for IHC (Autostainer, Dako) according to the manufacturer's guidelines. The slides were counterstained with haematoxylin.

## Competing interests

The authors declare that they have no competing interests.

## Authors' contributions

NW, NOD, MC and PD contributed substantially to conception, design, analysis and interpretation of the data. NW carried out experiments. SK sourced and scored clinical material. MH and PD carried out 2D DIGE MALDI-TOF MS analysis. PM supervised and coordinated proteomic studies. NW, NOD, MC and PD have been involved in drafting the manuscript and revising it critically for important intellectual content.

## Supplementary Material

Additional file 1 **Table S1**. Differentially expressed proteins identified in the comparison of Clone #3 and Clone #8. Click here for file
