# Supplementary material for: Identification of pancreatic cancer invasion-related proteins by proteomic analysis
Source: Proteome Sci. 2009 Feb 14;7:3. doi: 10.1186/1477-5956-7-3 (PMC2646716; doi:10.1186/1477-5956-7-3)
Supplement: Additional file 1 — Table S1. Differentially expressed proteins identified in the comparison of Clone #3 and Clone #8. [file 1477-5956-7-3-S1.doc]

Table S1: Differentially expressed proteins identified in the comparison of Clone #3 and Clone #8

| No. | Protein name | Protein AC number | Theoretical  pI/Mw | %  Coverage | Fold  change | Function |
| --- | --- | --- | --- | --- | --- | --- |
| 1 | Aldehyde dehydrogenase 1 (a) | gi|2183299| | 6.3/55.4 | 21.8 | 8.9 *** | Aldehyde dehydrogenase activity |
| 2 | Aldehyde dehydrogenase 1 (b) | gi|2183299| | 5.2/49.7 | 33.2 | 8.4 *** | Aldehyde dehydrogenase activity |
| 3 | Vimentin (a) | gi|57471646| | 5.2/49.7 | 37.9 | 5.5 *** | Cytoskeleton |
| 4 | Aldehyde dehydrogenase 1 (c) | gi|2183299| | 6.3/55.4 | 28.5 | 5.3 *** | Aldehyde dehydrogenase activity |
| 5 | Triosephosphate Isomerase (a) | gi|66360366| | 6.5/26.8 | 19.4 | 4.0 *** | Glycolysis |
| 6 | Vimentin (b) | gi|57471646| | 5.2/49.7 | 33.2 | 2.8 *** | Cytoskeleton |
| 7 | Vimentin (c) | gi|57471646| | 5.1/53.7 | 36.5 | 2.7 * | Cytoskeleton |
| 8 | Ubiquitin-activating enzyme E1 | gi|57209338| | 5.5/119 | 4.9 | 2.6 *** | Protease activity |
| 9 | Heat shock 70kDa protein 4 isoform a variant | gi|62087882| | 5.4/88.8 | 10.5 | 2.4 *** | Chaperone activity |
| 10 | Structure Of Human Muscle Pyruvate Kinase | gi|67464392| | 7.8/58.5 | 41.8 | 2.4 *** | Kinase activity |
| 11 | Elongation factor 1-alpha 1 (a) | gi|28848610| | 5.3/43.2 | 10.1 | 2.4 *** | Translation regulatory activity |
| 12 | Translation elongation factor 1 alpha 1-like 14 (b) | gi|15277711| | 9.1/43.3 | 12.1 | 2.2 *** | Translation regulator activity |
| 13 | Vinculin | gi|24657579| | 5.8/117.3 | 15.6 | 2.2 *** | Cytoskeletal protein binding |
| 14 | Glucosidase II | gi|2274968| | 5.7/107 | 11.3 | 2.2 *** | Hydrolase activity |
| 15 | ER-60 protein (a) | gi|2245365| | 5.9/57.2 | 26.9 | 2.0 *** | Isomerase activity |
| 16 | Stress-induced-phosphoprotein 1 (a) | gi|54696884| | 6.4/63.3 | 17.9 | 2.0 *** | Receptor signalling scaffold activity |
| 17 | Vimentin (d) | gi|57471646| | 5.2/49.7 | 18.1 | 2.0 *** | Cytoskeleton |
| 18 | EEF2 protein (a) | gi|33869643| | 6.5/58.2 | 17.6 | 2.0 *** | Translation regulator activity |
| 19 | Pyruvate kinase 3 isoform 1 variant | gi|62897413| | 8.8/58.5 | 44.8 | 1.9 *** | Energy pathway |
| 20 | Heat shock 70kDa protein 8 isoform 1 variant | gi|62897129| | 5.3/71.1 | 28.2 | 1.9 *** | Heat shock protein activity |
| 21 | Enolase 1 variant | gi|62897945| | 7.0/47.5 | 31.8 | 1.9 *** | Catalytic activity |
| 22 | Eukaryotic translation elongation factor1 alpha1 (c) | gi|62897525| | 9.3/50.5 | 16.5 | 1.8 * | Translation regulator activity |
| 23 | GARS protein | gi|12652637| | 8.2/85.4 | 19.7 | 1.8 *** | Ligase activity |
| 24 | Human rab GDI | gi|285975| | 5.9/51.1 | 31.7 | 1.8 *** | Auxiliary transport protein activity |
| 25 | KIAA0098 protein | gi|58257644| | 5.5/61.5 | 38.3 | 1.8 *** | Chaperone activity |
| 26 | Stress-induced-phosphoprotein 1 (b) | gi|54696884| | 6.4/63.3 | 11.6 | 1.8 *** | Receptor signalling scaffold activity |
| 27 | Cyclophilin A Complexed With 2-Thr Cyclosporin | gi|3659980| | 8.1/18.1 | 30.3 | 1.7 ** | Isomerase activity |
| 28 | Tubulin alpha 6 variant (a) | gi|62897609| | 5.0/50.5 | 18.9 | 1.7 * | Structural constituent of cytoskeleton |
| 29 | Heat shock 70kDa protein 8 isoform 1 variant | gi|62897129| | 5.6/53.6 | 41.4 | 1.7 *** | Heat shock protein activity |
| 30 | ER-60 protein (b) | gi|2245365| | 5.9/57.2 | 25.5 | 1.7 *** | Isomerase activity |
| 31 | MTHSP75 | gi|292059| | 6.0/74.1 | 17.5 | 1.6 *** | Chaperone activity |
| 32 | Beta-tubulin (a) | gi|338695| | 4.7/50.2 | 32.9 | 1.5 *** | Structural constituent of cytoskeleton |
| 33 | Translation initiation factor | gi|496902| | 6.1/47.1 | 27 | 1.5 *** | Translation regulator activity |
| 34 | Heat shock 70kDa protein 8 isoform 1 variant (b) | gi|62897129| | 5.3/71.1 | 30.5 | 1.5 ** | Heat shock protein activity |
| 35 | ATP5A1 protein | gi|34782901| | 8.4/54.6 | 36.2 | 1.5 * | Transporter activity |
| 36 | ACTG1 protein (a) | gi|17511847| | 5.3/42.1 | 40.3 | 1.5 *** | Structural constituent of cytoskeleton |
| 37 | Tubulin alpha 6 variant (b) | gi|62897609| | 5.0/50.5 | 28.7 | 1.4 *** | Structural constituent of cytoskeleton |
| 38 | Tubulin; beta polypeptide (b) | gi|18088719| | 4.7/50.1 | 33.1 | 1.4 ** | Structural constituent of cytoskeleton |
| 39 | Protein disulfide isomerase-related protein 5 | gi|1710248| | 5.0/46.5 | 29 | 1.4 *** | Chaperone activity |
| 40 | Tubulin alpha 6 variant (c) | gi|62897609| | 5.0/50.5 | 29.4 | 1.4 ** | Structural constituent of cytoskeleton |
| 41 | Alpha-tubulin | gi|340021| | 4.9/50.8 | 32.6 | 1.4 ** | Structural constituent of cytoskeleton |
| 42 | Tubulin beta (c) | gi|338695| | 4.7/50.2 | 35.4 | 1.4 ** | Structural constituent of cytoskeleton |
| 43 | Fragment Of Human Tryptophanyl-Trna Synthetase | gi|50513261| | 7.1/43.6 | 35.2 | 1.4 *** | Ligase activity |
| 44 | MDH2 | gi|49168580| | 9.4/36.0 | 49.7 | 1.4 ** | Catalytic activity |
| 45 | ACO2 | gi|49168620| | 7.2/86.2 | 11.9 | 1.3 * | Metabolic activity |
| 46 | Human Muscle Fructose 1; 6-Bisphosphate Aldolase | gi|4930291| | 8.8/39.7 | 27.8 | 1.3 ** | Ligase activity |
| 47 | Annexin I (b) | gi|442631| | 7.9/35.3 | 14.6 | 1.3 * | Calcium ion binding |
| 48 | EEF2 protein | gi|33869643| | 9.1/65.4 | 13.6 | 1.2 * | Translation regulator activity |
| 49 | heat shock 70kD protein 9B (mortalin-2) | gi|5123454| | 6.0/74.1 | 38.5 | 1.2 * | Chaperone activity |
| 50 | HSPC108 | gi|6841440| | 6.9/38.6 | 23.6 | -1.2 * | Auxiliary transport protein activity |
| 51 | Ubiquinol-cytochrome-c reductase complex core protein I | gi|731047| | 5.9/53.3 | 20 | -1.3 *** | Catalytic activity |
| 52 | Heat shock 70kDa protein 8 isoform 1 variant (c) | gi|62897129| | 5.3/71.1 | 18.3 | -1.3 ** | Heat shock protein activity |
| 53 | Profilin 1 | gi|30582841| | 8.7/15.2 | 24.3 | -1.4 ** | Cytoskeletal protein binding |
| 54 | Heat shock protein 60 (a) | gi|77702086| | 5.7/61.4 | 42.8 | -1.5 *** | Heat shock protein activity |
| 55 | Antiquitin (a) | gi|34783121 | 6.2/55.9 | 13.7 | -1.5 ** | Oxioreductase activity |
| 56 | ACTB protein (c) | gi|15277503| | 5.6/40.5 | 20 | -1.6 ** | Structural constituent of cytoskeleton |
| 57 | Glyceraldehyde-3-phosphate dehydrogenase (a) | gi|31645| | 8.4/36.2 | 16.1 | -1.9 *** | Catalytic/Glycolysis activity |
| 58 | Antiquitin (b) | gi|34783121 | 6.2/55.9 | 17.6 | -2.2 *** | Oxioreductase activity |
| 59 | Glyceraldehyde-3-phosphate dehydrogenase (b) | gi|31645| | 8.4/36.2 | 29.6 | -2.6 *** | Catalytic/Glycolysis activity |
| 60 | Keratin 18 | gi|12653819| | 5.4/48.0 | 37.7 | -2.9 *** | Cytoskeleton |

The theoretical isoelectric point (pI) and molecular weight (Mw) were calculated from the sequence of the protein in the database. Isoforms of the same protein are referred to as (a), (b) etc. The percentage coverage is the amount of the protein sequence covered by the matched peptides. Statistical analysis between replicates is referred to as; * *p* ≤ 0.05, *** *p* ≤ 0.01, *** *p* ≤ 0.005.

Function – Molecular function determined from the human protein reference database ([www.hprd.org](http://www.hprd.org/)).
